# Discrimination and Dignity Experiences in Prior Oral Care Visits Predict Racialized Oral Health Inequities Among Nationally Representative US Adults

**DOI:** 10.1007/s40615-023-01821-0

**Published:** 2023-10-17

**Authors:** Sarah E. Raskin, Madhuli Thakkar-Samtani, Morgan Santoro, Eleanor B. Fleming, Lisa J. Heaton, Eric P. Tranby

**Affiliations:** 1 L. Douglas Wilder School of Government and Public Affairs, Virginia Commonwealth University, 1001 W. Franklin Street, Richmond, VA 23284 USA; 2 Analytics and Data Insights, CareQuest Institute for Oral Health, Boston, MA USA; 3 University of Maryland School of Dentistry, Baltimore, MD USA

**Keywords:** Oral health disparities, Oral health equity, Discrimination, Structural racism, Microaggressions, Provider-patient interactions

## Abstract

Racism, an oppressive and fallacious sociopolitical hierarchy, is a fundamental cause of oral health inequities worldwide. Everyday discrimination is associated with worse self-rated oral health, toothache and adult tooth loss, and lower oral care utilization. Few studies examine discrimination or microaggressions *within* oral care settings or their effects on oral health outcomes. We adapted the seven-item Everyday Discrimination Scale to the oral care setting (EDSOC); developed a four-item Dignity in Oral Care Scale (DOCS); fielded them to a probability-based nationally representative sample of US households as part of the 2022 State of Oral Health Equity in America survey (SOHEA, *n* = 5682); and examined associations between EDSOC and DOCS scores and three outcomes: self-rated oral health, duration since last oral care visit, and planning for future preventive/routine oral care. Nearly, all EDSCOC and DOCS measures were significantly associated with oral health outcomes. Discrimination experience in dental settings had an additive effect on reporting fair/poor oral health and a suppressive effect on planning a future dental visit. Indignity experience doubled-to-quadrupled the likelihood of reporting fair/poor oral health, not having visited a dentist in 2 years, and not planning a future oral care visit. Racio-ethnically minoritized patients may experience the unjust double bind of resolving urgent dental or completing preventive services needs amidst being treated in a discriminatory manner or without dignity. Oral health stakeholders should invest more effort to understand relationships between racism and oral health outcomes and introduce evidence-based interventions to ultimately abolish this societal harm.

## Introduction

Racism, an oppressive sociopolitical hierarchy organized around fallacious and mutable interpretations of the physical expressions of genotypic variation, is a fundamental cause of health inequities [1–3]. Racism reflects and perpetuates societal “beliefs, values, and distribution of resources” [4] about the advantage-worthiness, or superiority, of one group over the inferiority of other groups. Like other forms of (often intersecting) oppression such as sexism, classism, homophobia, and xenophobia, racism aggresses at societal (structural), institutional (systemic), interpersonal, and internalized levels [4–8].^1^ These unjust and avoidable enactments of power plus privilege may be explicit, socially accepted, or codified, as in sanctioned, normativized, or willfully overlooked discrimination. Enactments of racism may also be more veiled, tacit, framed as deniable or ambiguous, or even minimally or un- recognizable *to the enactor*, as in implicit bias or microaggressions [10–13]. Toward the pursuit of health equity, justice, and liberation, *all* forms of racism and oppression must be addressed.

A developing literature documents the harmful effects of racism on oral health [14–19]. Experiencing racio-ethnic discrimination is associated with diminished oral health outcomes including poorer overall and self-rated oral health, greater oral health impairment including toothache and adult tooth loss, and lower oral health-related quality of life (OHRQoL) among Black women in the USA, older foreign-born Chinese Chicagoans, First Nations women in Canada, Indonesian workers in South Korea, and Australian adults who self-identify as racio-ethnically minoritized and/or Aboriginal [20–28]. Experiencing racio-ethnic discrimination is also associated with comparatively lower utilization of oral health care among Black women and children in the USA, Hispanic-Americans regardless of racial self-identification, and racially minoritized Brazilian adults, with variation based on frequency or intensity of discrimination experience, socioeconomic status, and severity of dental anxiety, which is itself associated with experiences of discrimination [20–23, 26, 27]. In one nationally representative survey of US adults, experiencing *emotionally impactful* racio-ethnic discrimination had a dampening effect on dental utilization, though discrimination experience itself did not [29]. Qualitative studies elaborate these findings by explicating how experiencing racio-ethnic discrimination disincentives dental care-seeking among Mexican-American and Mexican migrant parents in the USA [30–32], Aboriginal Australian parents [33], indigenous parents and caregivers in the Ecuadorian Amazon [34], and others.

The empirical literature on racism and oral health is characterized by some meaningful limitations that inhibit knowledge development and that impede translation of findings to inform practice. The majority of studies use discrimination as the independent variable, consistent with the broader literature that documents the health effects of *explicit* interpersonal racism and inadequately considers the effects of microaggressions [8, 16, 35, 36]. Most survey-based studies document the oral health effects of participants’ *extra-clinical* experiences of discrimination, often using overly broad measures such as single item measures of lifetime experience of discrimination [24, 25, 28, 37–39]. Among the scant research that examines minoritized individuals’ experiences of racio-ethnic discrimination *by* oral care providers, most studies document anticipated or simulated discrimination [30–34, 40–42]. One notable exception is Sokoto and colleague’s community-based work, which found that over one third of pregnant Black/African American women in central Appalachia have experienced racism in an oral health care setting, and that dental fear and anxiety itself, statistically predicted by experiencing racism in oral care, reduced dental utilization [27]. In addition, all of these studies examine associations between experiences of racism and current or recent oral health outcomes but fail to capture how experiencing racism informs future dental visiting, limiting optimal translation into practical application. To our best knowledge, no previous research has documented the oral health effects of patients’ experience of racio-ethnic discrimination in oral care settings among a nationally representative sample in the USA. In addition, no known studies have documented the oral health effects of patients’ in-clinic experiences of microaggressions or, following Sue and colleagues’ original theorization, “brief and commonplace daily verbal, behavioral, and environmental indignities, whether intentional or unintentional, that communicate hostile, derogatory, or negative racial slights and insults to the target person or group” [12].

Oral health equity research agenda urge nuanced examinations of intersectional oppression in dental research, practice, and education, emphasizing the development and testing of mid-range theories of how oppression—and liberation—transects structural, institutional, interpersonal, and internalized levels [15, 16, 43–47]. This knowledge gap inhibits the translation of systematically produced evidence into practice. For example, this lack of mid-range theories stalls the development and formalization of intersectional anti-racist curricula to acquaint dental students with the relationships between systemic, interpersonal, and internalized oppression and help translate it into their clinical practice and advocacy [48]. This study responds to these calls by documenting experiences of discrimination in oral care settings among a national sample of US adults. We adapted the Discrimination in Medical Settings Scale (DMSS), itself based upon Everyday Discrimination Scale, for oral care settings [49] and fielded it as part of the State of Oral Health Equity in America survey (SOHEA) [50]. In addition to documenting explicit instances of discrimination as enumerated in these validated and broadly used instruments, we developed and piloted in SOHEA a new Dignity in Oral Care Scale (DOCS) in order to document patients’ experiences of racio-ethnic microaggressions experienced in the oral care setting.

## Methods

### Design and Participants

We completed this cross-sectional study by fielding the SOHEA survey to a probability-based nationally representative sample of US households in January and February 2022 via telephone and internet, following informed consent. The second annual survey of its type, the 2022 wave was the first to include discrimination and (in)dignity indices. CareQuest Institute for Oral Health designed and funded the survey; National Opinion Research Center (NORC) at the University of Chicago administered the instrument to its AmeriSpeak® panel. We based the sampling frame on age, race/Hispanic ethnicity, education, and gender, with an additional sample of American Indian/Alaskan native participants. Inclusion criteria included being 18 years of age or older and residing in a household that had not previously enrolled. Exclusion criteria included being under age 18 or residing in a household with someone who had already responded. The sampling unit was 17,603, with a final sample size of 5682. The final weighted cumulative response rate was 4.0%, and the margin of error is 1.75%. This study was approved by WCG IRB, Work Order #1-1519349-1. We prepared this manuscript following the Strengthening the Reporting of Observational Studies in Epidemiology (STROBE) guidelines [51].

### Variables

#### Outcome Variables

We examined three self-reported oral health outcomes:

1. In general, how would you rate your oral health (state of your teeth, mouth, and gums)? (excellent, very good, good, fair, poor).
2. When was your last visit to a dentist? (less than 6 months ago, between 6 months and 1 year ago, more than a year and less than 2 years ago, more than 2 and less than 5 years ago, 5 or more years ago, never).
3. Do you plan on seeing an oral health provider in the next year for routine or preventive care? (yes, no, unsure).

#### Independent Variables

Primary independent variables comprised three categories:

1. *General experiences of discrimination.* Using binary measures, we asked participants:
  - Have you experienced discrimination as a result of any of the following in your lifetime: race/ethnicity, age, gender, religion, physical appearance, sexual orientation, language, other? (non-exclusive yes/no).
  - Do you experience discrimination on a weekly basis? (weekly yes/no).
  - Have you ever been denied health care or oral health care due to discrimination? (ever, yes/no).

In this paper, we primarily report on findings from items 1b and 1c.

2. *Experiences of prior year discrimination in oral care*. We adapted the Everyday Discrimination Scale [52], a widely validated and used scale, to the oral care setting (EDSOC) following Peek and colleagues’ example of adaptation to the medical setting [49]. We asked respondents to state how frequently the following statements had occurred in the last year:
  - You received poorer oral health care than others.
  - You felt that a dentist or oral health team member acted as if he or she thinks you are not smart.
  - You felt that a dentist or oral health team member acted as if he or she is afraid of you.
  - You felt that a dentist or oral health team member acted as if he or she is better than you.
  - You felt like your dentist or oral health team was not listening to what you were saying.
  - A dentist or oral health team member called you names or insulted you.
  - You felt that a dentist or oral health team member threatened or harassed you.
  We preserved the original scale’s five-point linear answer options (never, rarely, sometimes, mostly, always). The Cronbach’s alpha for the EDSOC was 0.88, and the measure of this variable was based on the total scale scores.
3. *Experiences of microaggressions in most recent dental visit*. We developed Dignity in Oral Care Scale (DOCS), a novel scale based on the literature on patients’ experiences of microaggressions in health care encounters [10–13, 36, 53], which we pilot tested prior to application to the full sample. We asked respondents to state how much they agreed with the following statements about their last oral health visit:
  - My oral health provider believed me when I reported my oral health needs, knowledge, and behaviors.
  - My oral health provider respected me.
  - I trusted the oral health provider I saw.
  - My oral health provider tried to make me feel comfortable and at ease.
  Answer options included: strongly agree, somewhat agree, neutral, somewhat disagree, and strongly disagree. The Cronbach’s alpha for the DOCS was 0.92, and the measure of this variable was based on the total scale scores.

#### Other Variables

Control variables included age, race, sex, education status, employment status, income, location (urban rural), dental insurance, and health insurance.

### Statistical Methods

We produced descriptive statistics for each variable in order to summarize the weighted frequencies and percentages for the independent variables, outcome variables, and control variables. For the purposes of analysis, we rendered variables on the discrimination (EDSOC) and (in)dignity (DOCS) linear scales into summary scores, from 0 to 35 and 0 to 20, respectively. We ran chi-square tests to examine bivariate relationships between both the EDSOC and DOCS scales and control variables. We then ran multivariable logistic regression models for each outcome variable by each primary independent variable, adjusting for control variables. Because the DOCS scale is a novel scale, we examined the variables separately in regression analysis. All variables of interest were included in the model, and those with *p*-values of *p* < 0.05 were considered significant. There was no evidence of multicollinearity that would bias results, with all VIF statistics being under 4. Sampling weights were applied to all analyses. We completed all analyses by using the Stata 14 statistical package [54].

## Results

The overall survey sample size was 5682 people, characterized by a balanced age distribution, of whom 51.2% self-identified as female (Table 1). Hispanic respondents comprised the largest proportion of racio-ethnically minoritized respondents (17%), with Black non-Hispanic respondents and Asian non-Hispanic respondents respectively comprising 12% and 5.3% of the sample. The majority of respondents were educated beyond high school (62.1%), employed or retired (77%), and urban-dwelling (71.6%). Slightly more than half of respondents reported an annual income of less than $60,000, with one quarter of respondents earning less than $30,000 each year. Proportionately more participants had primary health insurance coverage than primary dental insurance coverage (89.8% vs. 69.5%), with private insurance coverage as the primary mechanism for both and Medicare the second-most common source of health insurance but not dental. Among medically uninsured participants (*n* = 534), 58.2% had not been insured in more than 2 years. Among dentally uninsured participants (*n* = 1,680), 79.9% had not been insured in more than 2 years and one-third had never had dental insurance. Nearly three-quarters of participants rated their oral health as good, very good, or excellent (74.7%). More than two-thirds of participants had had a dental visit in the prior year (65%), and 84.8% planned to complete a preventive oral health visit in the next year.

**Table 1:** Sample descriptive statistics

	**Frequency (%)**
**Age:**	
18–29	1157 (20.4%)
30–44	1473 (25.9%)
45–59	1328 (23.4%)
60+	1724 (30.3%)
**Gender:**	
Male	2734 (48.3%)
Female	2935 (51.7%)
**Race:**	
White, non-Hispanic	3554 (62.5%)
Black, non-Hispanic	681 (12%)
Other, non-Hispanic	178 (3.2%)
Hispanic	968 (17%)
Asian, non-Hispanic	301 (5.3%)
**Place of birth:**	
In the United States	5191 (91.8%)
Outside the United States	463 (8.2%)
**Education:**	
Less than high school	546 (9.6%)
High school graduate or equivalent	1609 (28.3%)
Vocational/technical school/some college/associates	1538 (27.1%)
Bachelor’s degree	1167 (20.5%)
Post graduate study/professional degree	823 (14.5%)
**Employment:**	
Working-as a paid employee	2843 (50%)
Working-self-employed	434 (7.6%)
Not working	1301 (22.9%)
Retired	1104 (19.4%)
**Income:**	
Less than $30,000	1421 (25%)
$30,000 to under $60,000	1460 (25.7%)
$60,000 to under $100,000	1384 (24.4%)
$100,000 or more	1417 (24.9%)
**Urbanicity:**	
Rural	1030 (18.1%)
Suburban	581 (10.2%)
Urban	4071 (71.6%)
**Primary health insurance:**	
Private insurance	2791 (54.7%)
Medicaid	606 (11.9%)
Medicare	1270 (24.9%)
Military	137 (2.7%)
Other	296 (5.8%)
**If uninsured, last time had health insurance:**	
Within the last year	117 (21.9%)
1–2 years ago	106 (19.9%)
2–5 years ago	97 (18.2%)
5 or more years ago	125 (23.4%)
Never	89 (16.7%)
**Primary dental insurance:**	
Private	2781 (70.5%)
Medicaid	511 (13%)
Medicare advantage or supplemental plan	352 (8.9%)
Military	70 (1.8%)
Other	233 (5.9%)
**If uninsured, last time had dental insurance:**	
Within the last year	151 (9%)
1–2 years ago	186 (11.1%)
2–5 years ago	257 (15.3%)
5 or more years ago	560 (33.3%)
Never	526 (31.3%)
**Self-rated oral health:**	
Excellent	481 (8.5%)
Very good	1754 (30.9%)
Good	2009 (35.4%)
Fair	1022 (18%)
Poor	412 (7.3%)
**Last dental visit:**	
Less than 6 months ago	2605 (45.9%)
Between 6 months ago and 1 year ago	1083 (19.1%)
More than a year and less than 2 years ago	737 (13%)
More than 2 and less than 5 years ago	732 (12.9%)
5 or more years ago	460 (8.1%)
Never	61 (1.1%)
**How often do you think you should see an oral health provider:**	
Every 3 to less than 6 months	750 (8.5%)
Every 6 to less than 12 months	3881 (30.9%)
Every year to every other year	891 (35.4%)
Only when there is a problem	130 (18%)
Never	24 (7.3%)
Experience discrimination on a weekly basis:*	
Yes	239 (10.9%)
No	1852 (84.1%)
I’d rather not say	112 (5.1%)
Denied oral health care due to discrimination:**	
Yes	80 (3.7%)
No	2059 (94%)
I’d rather not say	52 (2.4%)
**Plan on seeing an oral health provider in the next year:**	
Yes	4807 (84.8%)
No	296 (5.2%)
Unsure	564 (10%)

*2203 out of 5682 responded

**2191 out of 5682 responded

### Outcome Results

More than 43% of participants had experienced discrimination in their lifetime (*n* = 2472), with race/ethnicity (18.7%), physical appearance (15.1%), and gender (14%) as the most frequently cited types of discrimination (Table not shown). Nearly 40% of the sample completed the questions “Do you experience discrimination on a weekly basis” and “Have you been denied oral health care due to discrimination.” Almost three times as many participants reported experiencing weekly discrimination or selected “I’d rather not say” as those who reported being denied oral health care due to discrimination (Table 1). Nine percent of Black respondents, 6% of those who selected “Other” race/ethnicity, and 4% of those who self-identified as multi-racial reported having been denied oral care in their lifetime due to discrimination, as compared with 3.7% of the overall sample. Hispanic and multi-racial respondents were overrepresented among those who would “rather not say.”

There was little differentiation in experiences of discrimination (mean EDSOC score = 2.19 (SD = 4.09) or indignities (mean DOCS score = 2.14 (SD = 3.20)) in the oral health care setting among respondents within some demographic categories, for example by gender, place of birth, or urbanicity (Table 2). Other respondent categories indicated more substantive variation across subgroups, with resonance between experiences of discrimination and experiences of indignity; scores are reported as means. More younger participants (18–44) reported experiencing discrimination (EDSOC score = 3.30 (SD = 4.97)) and less dignified treatment in oral health settings (DOCS score = 2.83 (SD = 3.39)) than did older participants (45+) (EDSOC scores = 1.62-1.13 (SD = 2.48–3.23); DOCS scores = 1.87–1.42 (SD = 2.98–2.69)). Participants who self-categorized with a racially/ethnically minoritized identity more commonly experienced discrimination (EDSOC scores = 2.71–3.23 (SD = 2.96–3.43)) and indignities in oral health settings (DOCS scores = 2.46–2.61 (SD = 2.96–3.28)), on average, than did white non-Hispanic participants (EDSOC score = 1.66 (SD = 3.51); DOCS score = 1.89 (SD = 3.11)), as did participants who identified with markers of a lower-class status (e.g., limited educational attainment, lower income, not working, and being insured by Medicaid) as compared with participants who identified with markers of a higher-class status (Table 2). Participants who reported never having health insurance reported the highest averages of being discriminated against (EDSOC score = 6.76 (SD = 7.00)) and being treated in an undignified manner (DOCS score = 5.05 (SD = 4.13)) in an oral health setting, while a similar pattern was not observed among those who previously had lost dental insurance (Table 2).

**Table 2:** Dignity and discrimination scale averages (standard deviation) by demographics

	Dignity scale (DOCS)*	***p*-value**	Discrimination scale (EDSOC)**	***p*-value**
**Age:**		< 0.001		< 0.001
18–29	2.83 (3.39)		3.30 (4.97)	
30–44	2.69 (3.57)		3.12 (5.02)	
45–59	1.87 (2.98)		1.62 (3.23)	
60+	1.42 (2.69)		1.13 (2.48)	
**Gender:**		< 0.001		0.008
Male	2.29 (3.27)		2.27 (4.19)	
Female	2.00 (3.13)		2.14 (3.98)	
**Race:**		< 0.001		< 0.001
White, non-Hispanic	1.89 (3.11)		1.66 (3.51)	
Black, non-Hispanic	2.61 (3.28)		3.18 (4.85)	
Other, non-Hispanic	2.52 (3.43)		2.71 (4.35)	
Hispanic	2.57 (3.41)		3.11 (4.78)	
Asian, non-Hispanic	2.46 (2.96)		3.23 (4.86)	
**Place of birth:**		0.005		< 0.001
In the USA	2.15 (3.23)		2.19 (4.10)	
Outside the USA	1.89 (2.80)		2.10 (3.80)	
**Education:**		< 0.001		< 0.001
Less than high school	3.24 (3.65)		3.69 (5.09)	
High school graduate or equivalent	2.79 (3.49)		2.94 (4.78)	
Vocational/technical school/some college/associates	2.16 (3.19)		2.11 (3.98)	
Bachelor’s degree	1.49 (2.82)		1.32 (2.93)	
Post graduate study/professional degree	1.07 (2.16)		1.20 (2.71)	
**Employment:**		< 0.001		< 0.001
Working-as a paid employee	2.10 (3.19)		2.17 (4.03)	
Working-self-employed	2.37 (3.37)		2.29 (4.30)	
Not working	2.81 (3.47)		3.30 (4.99)	
Retired	1.36 (2.62)		0.95 (2.16)	
**Income:**		< 0.001		< 0.001
Less than $30,000	3.21 (3.71)		3.75 (5.36)	
$30,000 to under $60,000	2.24 (3.10)		2.17 (3.92)	
$60,000 to under $100,000	1.81 (3.02)		1.68 (3.49)	
$100,000 or more	1.32 (2.57)		1.19 (2.66)	
**Urbanicity:**		0.02		< 0.001
Rural	2.27 (3.29)		2.43 (4.52)	
Suburban	2.26 (3.47)		2.09 (3.94)	
Urban	2.09 (3.14)		2.16 (3.99)	
**Primary health insurance:**		< 0.001		< 0.001
Private insurance	1.78 (2.95)		1.66 (3.10)	
Medicaid	3.12 (3.55)		3.50 (4.78)	
Medicare	1.71 (2.91)		1.71 (3.57)	
Military	1.41 (2.38)		2.24 (4.43)	
Other	2.54 (3.31)		2.30 (4.26)	
**If uninsured, last time had health insurance:**		< 0.001		< 0.001
Within the last year	3.38 (3.96)		3.98 (5.74)	
1–2 years ago	4.27 (3.72)		5.93 (5.69)	
2–5 years ago	4.41 (3.87)		4.85 (6.01)	
5 or more years ago	3.00 (3.72)		2.89 (4.74)	
Never	5.05 (4.13)		6.76 (7.00)	
**Primary dental insurance:**		< 0.001		< 0.001
Private	1.64 (2.89)		1.65 (3.43)	
Medicaid	3.26 (3.68)		3.99 (5.05)	
Medicare advantage or supplemental plan	1.74 (2.91)		1.58 (3.40)	
Military	1.57 (2.50)		1.56 (3.29)	
Other	2.37 (3.15)		2.46 (4.19)	
**If uninsured, last time had dental insurance:**		< 0.001		< 0.001
Within the last year	2.84 (3.37)		3.32 (4.63)	
1–2 years ago	2.76 (3.10)		3.92 (4.86)	
2–5 years ago	2.89 (3.41)		3.31 (5.54)	
5 or more years ago	2.35 (3.28)		1.73 (3.51)	
Never	2.95 (3.70)		2.65 (4.83)	

*Comprised of four questions. Scale ranges from 0 to 16

**Comprised of seven questions. Scale ranges from 0 to 28

### Main Results

Nearly all measures of discrimination and (in)dignity were significantly associated with the three oral health outcomes of interest (Table 3). Respondents who reported experiencing racial or ethnic discrimination in general or everyday settings on a weekly basis were almost 40% more likely to report their OH status as fair/poor (OR = 1.39; 95% CI = 1.18–1.64; *p* = 0.001) and to not have had a dental visit in the two preceding years (OR = 1.36; 95% CI = 1.14–1.62; *p* = 0.001), as compared with respondents who report not experiencing racial or ethnic discrimination on a weekly basis, after adjusting for covariates. Past year racio-ethnic discrimination experiences in the oral care setting had an accumulative effect on all oral health outcomes. With each additional experience of discrimination in an oral care setting in the prior year reported using an EDSOC measure, respondents were 58% more likely to report fair/poor oral health status (OR = 1.58; 95% CI = 1.43–1.77; *p* < 0.001)) and 37% more likely to report being unsure or not planning to attend a future routine or preventive dental visit (OR = 1.37; 95% CI = 1.21–1.55; *p* < 0.001) after adjusting for covariates.

**Table 3:** Oral health outcomes by discrimination and dignity in everyday and oral health care settings

	**Oral health outcomes**					
	**Oral health status: fair/poor**		**No oral health visit in the prior 2 years**		**Future dental visit: no/unsure**	
	OR (95% CI)	*p*-value	OR (95% CI)	*p*-value	OR (95% CI)	*p*-value
*Discrimination*						
Experienced racial/ethnic discrimination in unspecified/general settings on a weekly basis*	1.39 (1.18–1.64)	< 0.001	1.36 (1.14–1.62)	0.001	0.85 (0.68–1.06)	0.15
Experienced racial/ethnic discrimination in an oral health care setting in prior year**	1.58 (1.43–1.77)	< 0.001	1.16 (1.04–1.30)	0.009	1.37 (1.21–1.55)	< 0.001
Dignity***						
Felt believed: disagree/neutral in an oral health setting in prior year	2.12 (1.82–2.48)	< 0.001	3.51 (2.68–3.69)	< 0.001	3.32 (2.74–4.03)	< 0.001
Felt respected: disagree/neutral in an oral health setting in prior year	2.51 (2.13–2.96)	< 0.001	3.58 (3.02–4.25)	<0.001	3.62 (3.00–4.38)	< 0.001
Trusted OHP: disagree/neutral in an oral health setting in prior year	2.40 (2.04–2.82)	< 0.001	3.62 (3.06–4.29)	< 0.001	3.66 (3.03–4.42)	< 0.001
Felt comfortable: disagree/neutral in an oral health setting in prior year	2.44 (2.06–2.88)	< 0.001	3.98 (3.35–4.73)	< 0.001	3.32 (2.74–4.03)	< 0.001

*As compared with respondents who did not report racial or racial/ethnic discrimination

**A scale comprised of seven questions where scores range between 0 and 35

***As compared with respondents who agreed

All measures on the DOCS scale (DOCS) were meaningfully associated with all dental outcomes. Respondents’ experiences of being disbelieved in their dental complaint (OR = 2.12; 95% CI = 1.82–2.48; *p* < 0.001), disrespected (OR = 2.51; 95% CI = 2.13–2.96; *p* < 0.001), unable to trust their oral health provider to act in their best interest (OR = 2.40; 95% CI = 2.04–2.82; *p* < 0.001), or not made to feel comfortable (OR = 2.44; 95% CI = 2.06–2.88; *p* < 0.001) were associated with odds that were more than twice as great of reporting fair or poor oral health status as respondents who felt believed, respected, able to trust their oral health provider, and made to feel comfortable. Dignity measures were even more strongly associated with oral health visiting measures. Having not had an oral health visit in the prior 2 years was four times as common among participants who reported that they were not made to feel comfortable in the oral care environment (OR = 3.98; 95% CI = 3.35–4.73; *p* < 0.001). Participants’ experiences of feeling disrespected or not trusting their provider to act in their best interest were associated with odds that were more than three-and-a-half-times as great of reporting not having had an oral health visit in the prior 2 years (disrespect OR = 3.58; 95% CI = 3.02–4.25; *p* < 0.001; distrust OR = 3.62; 95% CI = 3.06–4.29; *p* < 0.001)) or to be planning a future preventive or routine care visit (disrespect OR = 3.62; 95% CI = 3.00–4.38; *p* < 0.001; distrust OR = 3.66; 95% CI = 3.03–4.42; *p* < 0.001)).

### Other Analyses

**Fig. 1:**
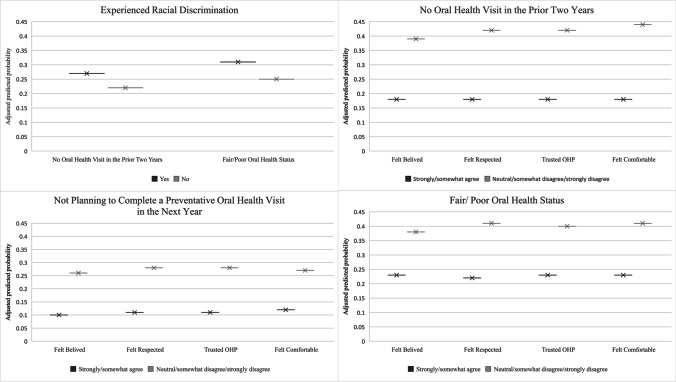
Adjusted predicted probabilities of oral health outcomes

Adjusted predicted probability (APP) results verified the strength of our primary results. Survey respondents who reported experiences of undignified treatment in the oral care setting had a higher APP of reporting poor or fair oral health status in the direction of the odds ratios (0.38–0.41) after controlling for covariates, as compared with respondents who experienced more dignified treatment (OR 0.22–0.23) (Fig. 1). Participants who experienced indignities in oral care settings had a much higher APP of not having had an oral health visit in the prior 2 years as compared to those who experienced more dignified treatment (0.39–0.44 vs. 0.18) and of not planning to complete a preventive oral health visit in the next 2 years as compared to those who experienced more dignified treatment (0.26–0.28 vs. 0.1–0.12) after controlling for covariates, also in the direction of the ORs (Fig. 1).

## Discussion

The deleterious effects of experiencing extra-clinical discrimination on oral health are increasingly documented among racio-ethnically minoritized individuals in communities globally and within the USA, with a burgeoning literature on the harmful oral health effects of discrimination within care settings [16, 20–27, 29–34, 37, 42]. Our study strengthens existing knowledge of the harmful oral health effects of everyday racio-ethnic discrimination by documenting these associations among a probability-based nationally representative sample and lending statistical verification to qualitative evidence of the deleterious effects of racism enacted within dental service settings on oral health outcomes [27, 30–33, 42]. We extend these findings by quantifying, to our best knowledge for the first time: the oral health effects of discrimination *within* oral care settings among a nationally representative sample in the USA; the effects of racism on preventive/routine oral care visits planning; and the oral health effects of microaggressions, or daily indignities, enacted in oral care settings. By adapting the Everyday Discrimination Scale to the oral care setting (EDSOC), which permits more precision than blunt binary measures of lifetime discrimination experience, we also document the additive effects of discrimination experiences on oral health outcomes. Moreover, in adding the DOCS measures, this study has documented how microaggressions and other indignities are experienced in oral care settings and their impact on oral health outcomes.

We found statistically significant associations between nearly all within-clinic experiences of discrimination and (in)dignity, and oral health outcomes including oral health status, prior dental visits, and planned preventive visits. We also found that these associations were substantive in the degree and the strength of their associations. Importantly, prior year in-clinic discrimination experience was more strongly associated with not planning to complete a future oral care visit than with not having received dental services in the prior year. These findings suggest that participants who experienced discrimination in an oral health setting may have sought dental care for urgent dental complaints, such as pain, infection, or repair of a broken restoration, *despite* awareness of the risk of discrimination, while foregoing for the sake of avoiding discriminatory or indignant interactions the very oral health services that could prevent need of an urgent appointment.

Findings on the harmful effects of microaggressions on oral health outcomes are independently meritorious. As measured by the DOCS, they may also explain some of the limitations of measurements of discrimination, namely the unique harms of “brief and commonplace… indignities,” [12] as compared with more explicit enactments. For example, being name-called or threatened by oral care providers (per EDSOC measurement) may be less pervasive due to changing social norms in general and the incorporation of patient-centered pedagogies into dental training in particular. However, research has documented the commonplaceness of veiled, tacit, implicitly biased, and ambiguous instantiations of interpersonal racism in other areas of health care such as pain management and reproductive health, as well as their negative effects on patients [55]. Our findings that respondents who feel disbelieved in their oral complaint, disrespected, not confident in oral care providers acting in their best interest, or not being made to feel comfortable are less likely to have completed a dental visit in the prior 2 years or plan to seek routine/preventive care, while unique in the oral health literature, are consistent with the broader literature on racism, health, and health care. The strength of association, in particular, demands our scientific attention to better understand these associations and to develop practices in oral care settings to make all patients feel affirmed, supported, and valued.

The well-documented and persistently lower oral care utilization rates among racio-ethnically minoritized adults in the USA, as compared with population-wide rates [56, 57], are commonly attributed to personal and structural resource constraints such as lack of dental insurance coverage, high cost of care, the exclusion of dental coverage from public insurance coverage, and geographic improximity to dental practices as a result of dental practices’ preferences for locating in higher wealth—and, therefore, proportionately more white—neighborhoods. These explanations are certainly meritorious, especially when contextualized as the result of political determinants of health such as the history of harmful racio-ethnically exclusionary policies in the USA. In our analysis, controlling for income, dental insurance coverage, and other factors associated with resource security indicates how microaggresssions threaten the timely oral care completion regardless of material resources. At the same time, we interpret our finding that the highest average reports of experiencing discrimination and indignity in dental settings were by participants who had never had health insurance through the context of comparatively lower rates of health insurance coverage and higher rates of insurance precarity among Black Americans, Hispanic Americans, and other racio-ethnically minoritized individuals. Consistent with other scholars who draw on intersectionality theory to study oral health disparities [6, 15, 23], we observe that even seemingly “race-neutral” variables in our study such as health insurance coverage likely mask an inequitable maldistribution of discrimination and indignity experiences—and their subsequent effects on oral health outcomes—along racio-ethnic lines. These findings indicate the importance of maintaining an intersectional anti-racism approach in studying dental access and utilization, and of framing advocacy to expand dental insurance coverage, reimbursement rates of public dental benefits, and supports and distributions of safety net clinics through a racial justice lens.

Findings from this study indicate the urgency of developing, testing, and routinizing evidence-based, anti-racist, oral care professional practices characterized by believing patients, respecting patients, and cultivating trust and comfort with patients [47]. Research in other health care fields find that patient-provider racial concordance can improve patients’ clinical outcomes and utilization as a result of patients feeling agentive and respected in their care [58–61]. Amidst many calls and some attempts in the last few decades to diversify the field of dentistry across the entire team [62–69], considering our findings through existing evidence on concordance indicates that an oral care workforce more representative of the population they serve may be key to delivering what Karbeah and colleagues describe as “historically- and culturally-safe” relationship-centered care (2022). Foremost is not only the enrollment, training, mentoring, and successful placement of historically underrepresented racial and ethnic (HURE) dental students in oral care professions, but also the introduction of HURE-related accountability into Commission on Dental Accreditation (CODA) standards used to guide student recruitment and admissions, faculty recruitment and retention, institutional culture, pedagogy, leadership development, and other holistic aspects of the educational environment [48, 70]. Our findings also lend support to proposed changes to CODA standards on predoctoral and specialized dental training, namely to familiarize *all* dental students with root cause analyses and the reflexive praxis necessary to overturn systemic oppression, approaches that have been favorably evaluated by dental students participating in institution-specific initiatives [66, 71, 72]. Vigorous anti-racist dental praxis should also form the basis of mandatory continuing education training for established practitioners.

### Limitations

Study limitations are as follows: the AmeriSpeak® panel’s representativeness of US households likely excludes residents experiencing housing insecurity, residents who are undocumented, and other historically disinvested populations. DOCS variables were developed from established theories of microaggressions and pilot tested with a representative sample, but did not undergo further testing.

## Conclusion

As the fields of oral health and dentistry increasingly theorizes, documents, and address racism as a structural determinant of oral health outcomes, evidence on the variety of enactments of racism are needed in order to understand the scope and characteristics of the problem and to identify with more precise opportunities for intervention. We find that enactments of both explicit discrimination and “subtler” microaggressions are associated with poorer oral health outcomes among a nationally representative sample of US adults, with the latter’s effects being more substantial. Oral health practitioners, educators, policymakers, thought leaders, and researchers should invest in more research to understand the nuanced relationships between racism at all levels—structural, systemic, interpersonal, and individual—and oral health outcomes, and identify and operationalize interventions to reduce and ultimately abolish this societal harm.

## Footnotes

1 The US Office of Management and Budget (USOMB) identifies race as comprising, at minimum, five categories (White, Black or African American, Asian, Hawaiian or other Pacific Islander, and American Indian or Alaska Native) and differentiates ethnicity as a binary category distinct of race (Hispanic or Latino or *not* Hispanic or Latino). USOMB categories of race and ethnicity are historically and contemporarily contested (see Sewell 2021) [9] but were used to collect the data analyzed here to optimize uniform standards guidance.
